# Late Childhood and Boarding Schools

**Published:** 1909-02-27

**Authors:** 


### Diseases of Children.

### LATE CHILDHOOD AND BOARDING SCHOOLS.

For practical purposes the periods of childhood may be divided into infancy, ending at two and a half years of age, when the temporary teeth are entirely erupted; early childhood, from the termination of infancy up to the commencement of the second dentition, say the seventh year; late childhood, lasting up to the commencement of puberty and sometimes called the prepubescent period; puberty or adolescence, from about the twelfth to the eighteenth year. Of course these are more or less arbitrary limits, not to be strictly applied, varying somewhat in different individuals, nationalities, and climates. There is no sharp line of demarcation; one passes more or less gradually into another, but occasionally about puberty the changes are extraordinarily rapid.

Boarding-school life should begin at eight to ten years of age. If a boy is physically strong and mentally sound, he should go to school at eight years of age. Should such a boy be particularly naughty or troublesome to manage at home, especially if his home is in London or any large town, he may be sent even at a year earlier in age. The advantages of companionship with his equals in age and strength, the routine and discipline of school, the open-air games and exercise far outweigh any special advantages ascribed to home influences and maternal love. It is nevertheless of the utmost importance that such children should go to comparatively small schools, specially arranged for boys of the age of seven to thirteen, and that on no account should they be sent to mixed schools for children of all ages. It is of dubious advantage bringing up children in mixed schools of both sexes, though there are no very serious objections to it during the prepubescent period. Possibly courtesy, and the habits included in what are known as good manners, may be more developed under such conditions, though in a properly managed school they are equally well taught without mixing the sexes. It is very doubtful whether it is advisable to encourage girls to enter into competition with boys at this age. The mere fact that the girl may sometimes outstrip the boy in fleetness of foot, more often still in intelligence and the rapid acquirement of knowledge in the form of lessons, is likely to give her a false estimate of her capabilities and lead her in later life to compete in the struggle for existence on a similar plane, at a time when her physical development renders it imperative that the education of intellect and body should be temporarily sacrificed to the requirements of sex.

Children at the age of eight are often supposed to be too young or too delicate for school. They are not, if they are sent to a school where there is a proper amount of supervision and care of the individual without coddling. In the stages of late childhood health is almost at its best. The disorders of early infancy have been avoided or recovered from. Many of the specific fevers may have been satisfactorily passed through. The records of our hospitals for children show an extremely low mortality at this period. The brain has almost reached its adult size, perception is acute, the mind restless and inquiring, the memory good and lasting, and the senses keen and alert. There is great endurance for short stretches, wonderful vitality and extraordinary activity. Watch a boy of this age, free and at large in the country, and one is astonished what an active existence he enjoys. The appetite is generally good, and a liberal supply of carbohydrate food is necessary to provide for this activity, as well as protein and fats for growth, heat, and nutrition. This is the feral stage of life, and is analogous to the age of maturity of primitive tribes of mankind in warm climates. It represents and is represented by the fundamental traits of savagery. It is the time of life when boys like to emancipate themselves from home influences, and become independent in thought and action. They like to make themselves caves or huts, to imagine themselves, and if possible to become, hunters, rovers, or pirates. Further, it must be noted that there is great plasticity of brain, sense organs, and muscles, and that at this age there is the greatest susceptibility to discipline and drill. On the other hand the psychical nature has not begun to unfold, or is only just beginning to do so. Sympathy, love, reason, morality, and religion are but slightly developed. Some boys exhibit more of these qualities than others as the result of careful teaching during home life, but even in these the development is little more than that of a more or less ill-learned lesson.

The judicious application of teaching and general management of boys at this age depends on a true understanding of the above factors in development. The rigid school discipline, routine lessons, obnoxious games, stodgy and ill-cooked meals may result in the production of an ordinary moderately educated boy who can pass into a public school, but does not in a real sense get the best results of education.

Teaching should be dogmatic and authoritative, with short hours and short periods of intense application. No reliance can be placed on interest or on reason, nor on work which is not done under observation. It should be objective as far as possible—a study of nature and mankind at first hand. It is doubtful whether, in any school, boys of this age have been taught to observe and study the manners, habits, and customs of their fellow creatures. Training should be external and mechanical; special attention being directed to drawing, to manual training, and to educating the voice in speech, singing, and languages. The feral instincts should not be rigidly suppressed. They may even be encouraged if they can be directed into safe channels, such as boy-scouting, the formation of summer camps, hunting expeditions on moors and in available woods for botanical or geological specimens, caterpillars, and grubs, and the study of bird and animal life. Nothing is more suited to this period than life in the woods and fields, in seaside camps, or even on the sea. The wilder and more undomesticated it is, within limits compatible with discipline and food requirements, the better it is for the physical and mental development of the child. One original observation is worth many accurately repeated lessons. Where such resources are not available benefit is derived from blood-and-thunder tales, stories of heroism or heroic virtues, and the simulation of these in charades or play acting. A good clean blood-and-thunder tale never did a boy any real harm. Above all it is essential that the hours of sleep should not be curtailed.

